# Plant Growth Regulators in Tree Rooting

**DOI:** 10.3390/plants11060805

**Published:** 2022-03-17

**Authors:** Federica Brunoni, Jesús Mª Vielba, Conchi Sánchez

**Affiliations:** 1 Laboratory of Growth Regulators, Faculty of Science, Palacký University and Institute of Experimental Botany, The Czech Academy of Sciences, 78371 Olomouc, Czech Republic; federica.brunoni@upol.cz; 2 Department of Plant Physiology, Instituto de Investigaciones Agrobiológicas de Galicia, Spanish National Research Council, 15780 Santiago de Compostela, Spain; jmvielba@iiag.csic.es

Trees are long-lived organisms with complex life cycles that provide enormous benefits both in natural and cultivated stands. They are essential components of forests and thus provide environmental, social, and economical goods and services [1]. Their influence extends beyond their key ecological roles as carbon sinks, soil protectors, habitats for other species, etc. Whether in natural forests, plantations or urban areas, they contribute in many ways to improve the quality of the ecosystems and have a significant impact in many fields such as the economy, climate change mitigation, health and social well-being, etc. [2]. Despite the relevance of trees, it is still not clear how many species of trees or woody plants exist, with recent estimates pointing to around 60,000, which represents around 20% of all angiosperm and gymnosperm plant species [3].

Woody and arborescent species usually present a high degree of plasticity in order to cope with the shifting conditions of the environment that they can face throughout their life cycles. Under the form of biotic or abiotic stress, trees have to deal with distinct constraints or limitations in their development, which are derived from their interaction with beneficial or pathogenic microorganisms and animals, the occurrence of flooding events, traumatic damages and wounds, drought seasons, etc. The roots of the trees and the resulting architecture of the root system are essential players in this plastic response needed to adjust tree growth to the shifting and challenging environment they must confront. The function of the roots must be sustained over time because, unlike annual plants, they have to perform their functions continuously to support tree growth and adaptation. From the primary root to the formation of lateral and adventitious roots (and root hairs), these organs work as the interface of the plant with the soil, ensuring the uptake of nutrients and water while at the same time providing physical support for the continuous growth of the aerial parts of the trees. The plasticity needed to cope with such demanding scenario is provided, at least partly, by the activity of the different phytohormones.

Acting at minute concentrations, phytohormones are chemical compounds that enable the transduction of external and internal cues to adapt plant growth to the ongoing conditions. In root development, the plastic behaviour shown by plants is a consequence of the channelling effect of phytohormones on the potential responses encoded by the genome and the epigenome that drives towards the most favourable responses to ensure plant survival and optimal growth. Phytohormone signalling during the modulation of root growth has long been studied and the role for specific regulators has been established, particularly in model crop species. Auxin acts as the master regulator of rooting, governing the organization of the root meristem and the processes of cell division and differentiation that give rise to new tissues. Various reports suggest that auxin-mediated events, which are key to establishing basic body organization and postembryonic organogenic processes, are of similar importance in both angiosperms and conifers (reviewed in [4]). In vegetative propagation of woody cuttings, an external source of auxin is usually needed to induce rooting, and the quality of the rooted cuttings is strongly affected by the concentration of exogenous auxin treatment [5,6]. However, the role played by other phytohormones is gaining attention, with each one of them showing significant influence in specific processes of root growth. For instance, cytokinin activity is essential for the maintenance of the root meristem, while a gibberellin gradient between different developing zones of the root guarantees continuous growth. Brassinosteroids and abscisic acid have also been shown to directly influence root development.

However, phytohormone analysis in tree rooting is an underrepresented area of study. Phytohormones might be present in the plant tissues at extremely low levels, and their homeostasis results in the presence of many structurally related but different compounds, thus increasing the need for specific and sensitive analysis. Moreover, root tissues are a complex chemical matrix, which increases the technical hurdles inherent to any plant extraction method.

Historically, phytohormone content analysis relied on chromatographic methods and antibody-based assays such as ELISA. Though these methods have proven extremely useful, they may not present enough sensitivity to detect the low concentration of these chemical compounds, the ability to distinguish closely related compounds, or they might fall short to detect the complete landscape of chemicals related to a specific phytohormone signalling route. Nonetheless, the popularization of mass spectrometry techniques provides the means for accurate quantification and precise identification of plant metabolites independently of the original matrix. Based on ultra-high-performance liquid chromatography–tandem mass spectrometry (UHPLC-MS/MS), Šimura et al. [7] developed a protocol for the extraction and quantification of more than one hundred phytohormone-related compounds, including auxins, cytokinins, gibberellins, abscisates, brassinosteroids, and jasmonates. This protocol enables the accurate characterization of specific hormone-profiles in plants. Moreover, extremely low amounts of samples are needed for the analysis, thus easing the study of differential concentrations of phytohormones within neighbouring tissues. MS-based quantification was used by Noah et al. [8] to determine the content of auxin and cytokinin metabolites in the initial stages of the development of the hypocotyl and primary root of cacao (*Theobroma cacao*). This analysis allowed the authors to track specific gradients of these two phytohormones in the different developing zones of the root, while also detecting the presence of amide-linked indole-acetic acid metabolites as the predominant form of auxin catabolite, rather than the oxidized by-products found in other species [8]. 

Optimization of the extraction protocol linked to the UHPLC-MS/MS application has allowed for analysing strigolactones in root tissues [9]. Using a similar approach, the characterization of the effect of strigolactones on the root system architecture and phytohormone content has been accomplished in cherry rootstocks [10]. Strigolactones treatment increased lateral root numbers and density, probably because of its modulation of the content in auxin, abscisic acid, and gibberellins. Combined with a transcriptomic analysis, it was revealed that genes related to several phytohormone-signalling pathways showed differential expression. Therefore, strigolactones emerge as a relevant factor in the modulation of root system architecture, at least in cherry tree [10]. In a nice example of phytohormone-driven integration of external information, the analysis of hormone content by means of UHPLC-MS-MS in bending roots of poplar showed the presence of specific gradients of auxin, cytokinin, and abscisic acid within the cambium region and surrounding areas. These gradients govern the developmental processes that lead to the formation of compression wood in these roots, a relevant adaptive trait in the response to mechanical forces [11]. Further analysis revealed the different behaviour of stems and roots of poplar subjected to the same kind of mechanical stress, with the gradients of auxin and cytokinins significantly influencing the process [12]. These results highlight the need for specific analysis of physiological responses in tree roots. Auxin and its metabolites were quantified to investigate indole-acetic acid (IAA) homeostatic regulation in conifers [13]. IAA metabolite formation was evaluated in root tissues under steady-state conditions and after perturbation of IAA homeostasis. This study showed that a diversification of IAA inactivation mechanisms occurred in conifers as oxIAA does not contribute considerably to maintaining IAA homeostasis in Norway spruce (*Picea abies*), as well as in other conifers such as Scots pine (*Pinus sylvestris*) and lodgepole pine (*Pinus contorta*) in contrast to Arabidopsis. Moreover, the irreversible conversion of IAA to IAAsp and IAGlu seems to act constitutively in steady-state conditions and to be the main pathway induced in response to perturbation of the IAA content, to maintain IAA homeostasis in conifers [13]. Different compounds can be used to improve the rooting responses in trees, particularly the formation of adventitious roots. A few examples show that these compounds might exert their activity in the process by modifying the phytohormone content. *Malus prunifolia* rootstocks are widely used for the vegetative propagation of apple trees. During the development of adventitious roots, exogenous application of the polyamine spermidine significantly improved the rooting response of the stems. ELISA-based analysis of the phytohormone content in response to spermidine revealed that it modulated the levels of auxins, gibberellins, and jasmonic acid, which resulted in changes in expression of several rooting-related genes [14]. In the same species and with the same method of quantification, it was found that melatonin treatment enhanced several parameters of the rooting process, including root number, root volume, and the surface area of the roots. The authors found that melatonin altered the profiles of several phytohormones, increasing auxin, cytokinin, and gibberellins and lowering abscisic acid two days after the beginning of the treatment. A cross-talk between melatonin and auxin might underlie these results [15]. The role of phytohormones during adventitious root initiation (ARI) was studied in de-rooted Norway spruce seedlings in response to different light conditions [16]. The authors showed that in contrast to white light, red light promoted ARI likely by reducing jasmonate (JA) and JA-isoleucine biosynthesis and repressing the accumulation of isopentyl-adenine-type cytokinins [16]. Despite the few examples shown above, there is an urgent need for more analyses in this field that might help to establish clear relations between phytohormone content and rooting responses in trees.

Epigenetic mechanisms are an essential tool for the adaptation of transcriptomic responses to changes in the environment, and are thus a key element to provide plasticity. Chromatin remodelling mechanisms and phytohormone signalling routes are two powerful systems to integrate information and render improved responses. Both systems seem to interact and even rely on each other, with specific chromatin remodelers governing many responses of particular phytohormones, while these compounds exert control on the expression of those remodelers according to the results available, mainly from Arabidopsis [17,18]. However, no clear picture of these interactions has emerged yet, and to our knowledge, no such studies have been developed in the analysis of tree rooting. 

The interactions between phytohormones signalling pathways occur at many levels, with the specific ratios among them establishing particular expression profiles for the same or different genes. MicroRNAs, 21–22 nucleotide hairpin-derived RNAs, exert a post-transcriptional control on the expression of their target genes, and behave as bridges between the signalling routes of phytohormones. MicroRNAs establish regulatory circuits in their interaction with phytohormones, mainly auxin, to govern root developmental processes (reviewed in [19]). Limited examples of such relations in tree or woody species were found. In *Malus xiaojinensis*, a regulatory hub comprising auxin and miRNA156 was shown to significantly influence the adventitious rooting process [20], while in grapevine a miRNA-derived peptide that is believed to regulate the expression of several GRAS transcription factors positively influences adventitious rooting [21]. The development of these studies and its combination with phytohormone analysis will provide the tools for more accurate breeding strategies.

In order to have a complete analysis of the root systems, imaging techniques must be used to entirely characterize root developmental processes. These techniques are becoming increasingly popular and have driven to the development of the so-called “phenomics”, although its application in the analysis of roots is relatively new. Different techniques are used to analyse root phenotypes, such as X-ray computed tomography, magnetic resonance, ground penetrating radar, etc. (reviewed in [22]). By means of electric resistivity tomography, the response of citrus tree roots was analysed under different irrigation regimes, showing clear differences in the growth direction of the roots [23]. Indeed, combination of imaging techniques with genetic studies might enhance our understanding of root development and help to identify new candidate genes involved in this process. In Arabidopsis, an integrated rhizotron imaging and GWAS analysis has allowed for the identification of genes that might be directly related to root system architecture [24]. However, examples in tree species are scarce at present. Exploitation of these techniques for woody species and the integrated analysis of phytohormone content might be a useful tool to develop tailored protocols for the optimization of tree cultivation.

By editing this Special Issue, our aim was to compile relevant studies concerning an underrepresented area of study: the analysis of the relationship between hormones and root growth in trees. We firmly believe that this is a field of outstanding relevance. Molecular analysis in tree and woody species analysis is always troublesome, and the knowledge gathered so far is still limited when compared to model species and relevant crops. Furthermore, results might not always be transferable between species, hindering the development of conceptual models. Despite the technical limitations that researchers might encounter when adopting forest and woody species as model systems, the knowledge gathered will certainly provide a basis for a more sustainable and improved manage of tree plantations and natural populations. Moreover, efforts in this direction are worth considering the current climate change scenario and the growing need to exploit the full potential of tree species. We encourage researchers to tackle these analyses, as the benefits in the long-term will certainly improve the profitability of trees from the economic and ecological perspectives.
